# Novel Heterozygous Mutation in *NFKB2* Is Associated With Early Onset CVID and a Functional Defect in NK Cells Complicated by Disseminated CMV Infection and Severe Nephrotic Syndrome

**DOI:** 10.3389/fped.2019.00303

**Published:** 2019-07-30

**Authors:** Alejandra Aird, Macarena Lagos, Alexander Vargas-Hernández, Jennifer E. Posey, Zeynep Coban-Akdemir, Shalini Jhangiani, Emily M. Mace, Anaid Reyes, Alejandra King, Felipe Cavagnaro, Lisa R. Forbes, Ivan K. Chinn, James R. Lupski, Jordan S. Orange, Maria Cecilia Poli

**Affiliations:** ^1^Clínica Alemana de Santiago, Facultad de Medicina Clínica Alemana-Universidad del Desarrollo, Santiago, Chile; ^2^Clínica Las Condes, Santiago, Chile; ^3^Hospital Padre Hurtado, Santiago, Chile; ^4^Section of Immunology, Allergy and Rheumatology, Department of Pediatrics, Center for Human Immunobiology, Baylor College of Medicine, Texas Children's Hospital, Houston, TX, United States; ^5^Department of Molecular and Human Genetics, Baylor College of Medicine, Houston, TX, United States; ^6^Human Genome Sequencing Center, Baylor College of Medicine, Houston, TX, United States; ^7^Division of Immunogenetics, Department of Pediatrics, Morgan Stanley Children's Hospital of New York Presbyterian, Columbia University Irving Medical Center, New York, NY, United States

**Keywords:** primary immunodeficiency, NF-κB2, common variable immunodeficiency (CVID), nephrotic syndrome, systemic cytomegalovirus, pituitary deficiency, NK cell deficiency

## Abstract

Nuclear factor kappa-B subunit 2 (NF-κB2/p100/p52), encoded by *NFKB2* (MIM: 164012) belongs to the NF-κB family of transcription factors that play a critical role in inflammation, immunity, cell proliferation, differentiation and survival. Heterozygous C-terminal mutations in *NFKB2* have been associated with early-onset common variable immunodeficiency (CVID), central adrenal insufficiency and ectodermal dysplasia. Only two previously reported cases have documented decreased natural killer (NK) cell cytotoxicity, and little is known about the role of NF-κB2 in NK cell maturation and function. Here we report a 13-year-old female that presented at 6 years of age with a history of early onset recurrent sinopulmonary infections, progressive hair loss, and hypogamaglobulinemia consistent with a clinical diagnosis of CVID. At 9 years of age she had cytomegalovirus (CMV) pneumonia that responded to ganciclovir treatment. Functional NK cell testing demonstrated decreased NK cell cytotoxicity despite normal NK cell numbers, consistent with a greater susceptibility to systemic CMV infection. Research exome sequencing (ES) was performed and revealed a novel *de novo* heterozygous nonsense mutation in *NFKB2* (c.2611C>T, p.Gln871^*^) that was not carried by either of her parents. The variant was Sanger sequenced and confirmed to be *de novo* in the patient. At age 12, she presented with a reactivation of the systemic CMV infection that was associated with severe and progressive nephrotic syndrome with histologic evidence of pedicellar effacement and negative immunofluorescence. To our knowledge, this is the third NF-κB2 deficient patient in which an abnormal NK cell function has been observed, suggesting a role for non-canonical NF-κB2 signaling in NK cell cytotoxicity. NK cell function should be assessed in patients with mutations in the non-canonical NF-κB pathway to explore the risk for systemic viral infections that may lead to severe complications and impact patient survival. Similarly NF-κB2 should be considered in patients with combined immunodeficiency who have aberrant NK cell function. Further studies are needed to characterize the role of NF-κB2 in NK cell cytotoxic function.

## Background

Common variable immunodeficiency (CVID) is one of the most frequently diagnosed symptomatic primary immunodeficiencies. Clinical and immunophenotypic manifestations are highly heterogeneous and it most typically presents clinically as recurrent infections, pulmonary inflammation with or without bronchiectasis, and sometimes associated with autoimmune manifestations such as granulomatous disease and lymphoproliferation (1). Characteristically, there is B cell dysfunction that results in impaired antibody production and hypogammaglobulinemia; and up to one third of the patients may have an associated T cell defect. The underlying genetic mechanisms for CVID have been elucidated in <10% of cases and more than 20 genetic causes for CVID have been described (1–3). NF-κB transcription factors are essential for the immune response, and mutations in NF-κB transcription factors and their regulators result in primary immunodeficiency and autoimmunity (4, 5). The non-canonical NF-κB pathway is critical for B cell survival, differentiation into plasma cells and isotype class switching as well as dendritic cell activation (6, 7). A number of mono- and biallelic mutations in this pathway have been identified among CVID patients (i.e., *BAFFR*, TACI*, TNFSF12, TRAF3, NIK, NFKB2*). Interestingly, in the case of *TACI/Taci* locus, the association of both heterozygous and biallelic variations with AD/AR CVID disease trait, suggest subtle gene dosage perturbations might underlie this phenotype (8).

Nuclear factor kappa-B subunit 2 (NF-κB2) is composed of p100 protein, a central component of the non-canonical NF-κB pathway that also serves as an inhibitor of the canonical pathway. The p100 protein is activated by phosphorylation of its C-terminal serine residues (Ser 866, Ser 870), triggering its breakdown to p52, that is then translocated into the nucleus (7). The association between CVID and central adrenal insufficiency had been previously recognized as Deficient Anterior pituitary with Variable Immune Deficiency (DAVID) syndrome (9). Later, heterozygous C-terminal mutations in *NFKB2* (MIM 164012) were determined to cause early onset CVID with variable association with central adrenal insufficiency, ectodermal dysplasia, and autoimmunity (10–16). The immune phenotype of these patients is characterized by profound B cell deficiency and defects in peripheral T cell proliferation and differentiation (12, 17). Decreased natural killer (NK) cell function has also been reported (15, 18).

Here we describe a female patient with a novel heterozygous nonsense mutation in *NFKB2* presenting with early onset CVID, ectodermal dysplasia, subclinical adrenal insufficiency and functional NK cell deficiency with overwhelming systemic CMV infection, uniquely associated with severe nephrotic syndrome. This case report highlights the relevance of NK cell function in prognosis and suggests that functional NK cell evaluation could impact treatment strategies in these patients.

## Case Presentation

**Figure 1:**
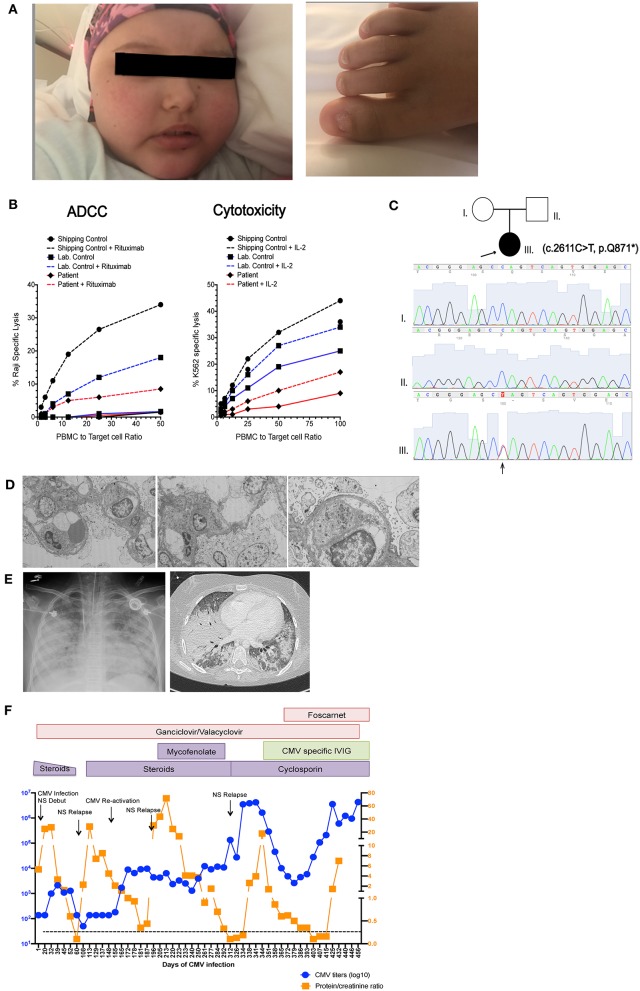
Patient clinical presentation. **(A)** Left picture shows the face of the patient with alopecia totalis, edema and Cushingoid facies due to prolonged steroid treatment. Right picture shows trachyonychia of the toenails. **(B)** Pedigree of the family showing two healthy non-consanguineous parents and the patient (arrow) with c.2611C>T mutation in *NFKB2*. Sanger tracings of each individual in the family are presented. **(C)** NK cell cytotoxic function measured by ^51^Cr release assay. Left graph shows antibody dependent cellular cytotoxicity (ADCC) measured against Raji B cell targets in in the presence (dashed line) and absence (solid line) of rituximab. Right shows natural cytotoxicity against K562 targets in the presence (dashed line) and absence (solid line) of IL-2 stimulation. Due to sample availability and transport limitations this demonstrates a single assay performed in triplicate. **(D)** Electron microscopy of renal biopsy showing pedicellar effacement and few mesangial deposits **(E)** Left picture shows a chest X ray and chest CT scan taken during the patient's final stage of disease showing multiple bilateral foci of alveolar compromise with extensive consolidation of bilateral basal lobes, superior left lobe and medial lobe. **(F)** Graphic representation showing variations in CMV titers in blood (in blue) and proteinuria (in orange), Black arrows indicate nephrotic syndrome (NS) reactivations. Immunosuppressive and antiviral treatments are indicated in colored boxes as they were administered during the course of disease.

A female patient born to non-consanguineous parents, with no family history of immunodeficiency or endocrine disorders was asymptomatic until 2 years of age when she presented with hair loss progressing to alopecia universalis, trachyonychia (sandpapered, rough nails with accentuated linear ridges), psoriatic-like dermatitis and atopic dermatitis. Facial and dental anomalies were not detected (Figure 1A). Subsequently, she developed recurrent bacterial upper and lower respiratory infections and immunologic evaluation at 6 years of age showed hypogammaglobulinemia (IgG 180 mg/dl, IgA< 4 mg/dl, IgM 4 mg/dl), low B cells with absent memory B cells and non-protective antibody titers to tetanus and pneumococcal vaccines consistent with CVID with absent B cells. T and NK cell numbers were normal, but T-cell proliferation to phytohemagglutinin (PHA) was decreased (Table 1).

**Table 1:** Patient immunophenotyping.

**Age (years)**	**10**	**11**	**11.5**	**12**	**12.5**	**Reference range**
**Lymphocyte populations % (Absolute)**						
CD3	95 (5025)	91 (5002)	89 (6998)	85 (974)	86 (877)	43–76%
CD4	69 (3615)	74 (4057)	62 (4941)	54 (640)	46 (464)	23–48%
CD8	24 (1263)	19 (1042)	23 (1858)	28 (329)	34 (345)	14–33%
CD19	1.7 (89)	2 (110)	2.8 (215)	5 (56)	4.6 (47)	14–44%
CD19+CD27+Memory B cells	0	0	0	0	0	1.5–4%
CD16/56	2.8 (147)	4 (219)	7.4 (577)	6.2 (69)	7.4 (75)	4–23 %
CD4/CD8 Ratio	2.8	3.9	2.6	1.9	1.3	0.9–2.9
T cell proliferation to PHA	50% Decrease	ND	Borderline low	ND	ND	

*ND, not determined*.

The patient remained free of significant infections on monthly intravenous immunoglobulin (IVIG) replacement until 9 years of age, when she developed CMV pneumonia that was successfully treated with intravenous ganciclovir, achieving negative CMV polymerase chain reaction (PCR) testing on follow up. After this first CMV infection she developed recurrent upper and lower respiratory infections despite immunoglobulin replacement and required antibiotic prophylaxis. CMV infection could have been a feature of her depressed T cell function but also raised the possibility for a functional NK cell abnormality. Hence, NK cell function was assessed by chromium release assay using K562 target cells, which demonstrated decreased NK cell cytotoxicity with poor improvement with IL-2, as well as decreased antibody-dependent cell cytotoxicity (Figure 1B).

After obtaining informed assent by the patient and informed consent from her parents, trio Whole Exome sequencing (WES) was performed and identified a *de novo* heterozygous c.2611C>T, (variant to total reads 123/221 = 0.55) p.Gln871^*^ variant in *NFKB2* (NM_001077494) leading to a premature stop codon in the C-terminal portion of NF-κB2. WES results at this locus and the *de novo* nature of the variant allele were confirmed by orthogonal Sanger sequencing (Figure 1C). NMDEscPredictor (19) was used to determine if this variant would be predicted to escape nonsense mediated decay (NMD). Similar to other C-terminal *NFKB2* variants (Table 2) Gln871^*^ is predicted to escape NMD and likely result in translation of a truncated protein. This variant is not present in ExAC, gnomAD, the 1000 Genomes Project or other personal genomes from ~9,000 subject samples studies by WES within our internal Center for Mendelian Genomics (CMG) database; therefore we are interpreting it as a novel disease-causing rare variant presumably loss of function (LoF) allele. The ExAC calculated probability of loss of function intolerance (pLi) for this gene is 1, meaning that it is unlikely to tolerate LoF. No other known primary immunodeficiency mutations or potential candidate gene were identified in this patient. With this result, pituitary function was assessed and studied by objective laboratory analyses that demonstrated reduced serum cortisol and adrenocorticotropic hormone (ACTH) levels; thus glucocorticoid replacement was initiated. Other pituitary hormones were within normal limits. Autoantibody testing, including antinuclear antibodies (ANA), anti-neutrophil cytoplasmic antibodies (ANCA), anti-adrenal and anti-thyroid antibodies were all negative.

**Table 2:** *NFKB2* mutations.

**Mutation**	**Infections**	**Immune phenotype**	**Autoimmunity**	**Endocrine abnormalities**	**References**
c.1252G>T (p.Glu418^*^) GR GoF	Recurrent sinopulmonary infections. Lymphoproliferation.	Low IgG, IgA and IgM, poor antibody response Low. T, B, and NK numbers, absent memory B cells NK function NA	Rheumatoid arthritis	-	(20)
c.2557C>T (p.Arg853^*^)	VRIs, pneumonias, sinusitis, otitis media, candida infection	Low IgG, IgA and IgM, low memory B cells, poor antibody response, decreased NK function variable among patients with same mutation.	Alopecia universalis, trachyonychia, anti-thyroid antibodies psoriasiform dermatitis^*^	Adrenal Insufficiency	(10, 13, 18, 21)
c.2556_2563del (p.Arg853Alafs^*^29)	Recurrent respiratory infections	Low IgG, IgA and IgM	No ectodermal features	Adrenal Insufficiency GH deficiency	(13)
c.2563A>T (p.Lys855^*^)	Recurrent respiratory infections	Low IgG, IgA and IgM, low t, B and NK cells, poor antibody response, low NK function	Alopecia universalis, trachyonychia, nephrotic syndrome	Adrenal Insufficiency	(15)
c.2564delA (p.Lys855Serfs^*^7)	Recurrent respiratory infections, sinusitis, peumonias, mucocutaneous candidiasis, recurrent herpes labialis	Low IgG, IgA, and IgM. Normal T and NK cells, low/normal B cells, Low memory B cells. NK cell function NA	Alopecia, trachyonychia, vitiligo	Adrenal Insufficiency	(10)
c.1903C>T (p.Arg635^*^) ARD GoF	Recurrent sinopulmonary infections, PJ pneumonia, recurrent thrush, warts. EBV and CMV viremia Bronchiectasis, recurrent herpes labialis in one patient. Lymphoproliferation.	Low IgG, IgA and IgM. Low CD4 T cells, low B cells. NK function NA		-	(20)
c.2593_2600del (p.Asp865Valfs^*^17)	Chronic sinusitis, bronchiectasis. Varicella infection in one patient.	Low IgG, IgA and IgM. Low B cells and memory B cells, low Tfh cells.	No ectodermal features	Adrenal Insufficiency	(14)
c.2594A>G (p.Asp865Gly)	No infections	Low IgG, IgA and IgM. Low B cells	Alopecia in one not in another	Adrenal Insufficiency in some patients	(11, 13)
c.2596A>C (p.Ser866Arg)	Molluscum contagiosum	Low IgG, IgA and IgM Normal T, B and NK numbers, absent memory B cells Protective antibody titers. NK function NA		Adrenal Insufficiency GH deficiency	(20)
c.2600C>T p.Ala867Val	No infections	Low IgG, IgA and IgM.	No ectodermal phenotype	Adrenal Insufficiency	(13)
c.2598insT (p.Ala867Cysfs^*^19)	Recurrent sinopulmonary infections	Low IgG, IgA and IgM, increased CD4/CD8 ratio, low memory B cells, poor antibody response, NK cell function NA	Alopecia universalis, trachyonychia	-	(12)
c.2611C>T (p.Gln871^*^)	Recurrent sinopulmonary infections, systemic CMV infection, PJ pneumonia.	Low IgG, IgA and IgM, low memory B cells, poor antibody response, decreased NK cell function	Alopecia	Adrenal Insufficiency	Current report

*This table summarizes the findings in CVID patients with NFKB2 mutations. Each mutation may have been found in more than one patient*.

* *Psoriasiform dermatitis was observed in the two patients reported with this variant. GR, glycine rich region; ARD, ankyrin repeat domain; GoF, gain of function; NA, not assessed*.

At 12 years of age the patient was admitted with gastroenteritis, generalized intestinal edema and severe nephrotic syndrome progressing to acute renal failure. Quantitative CMV PCR was positive >2 × 10^6^ copies/ml in blood and positive in a renal biopsy (not quantified in tissue). Renal histology showed focal-segmental glomerulosclerosis with extracapillary hypercellularity and mild tubulointerstitial atrophy. Ultrastructural findings showed complete pedicellar effacement characteristic of minimal change nephropathy and no intracellular inclusions or antibody deposits were demonstrated on cytopathology (Figure 1D). Immunofluorescence was faintly positive for C3 and IgG and negative for C1q, IgA, IgM, and fibrin. Exome files were screened for pathogenic podocin mutations that can be associated with nephrotic syndrome, and no pathogenic variants were identified. Interestingly, elevations in CMV titers in blood seemed to precede nephrotic syndrome relapses and correlate with increases in proteinuria (Figure 1F). Due to chronic systemic CMV infection and severe nephrotic syndrome with acute renal failure, she required intermittent critical level care and treatment with systemic steroids. She also received IVIG, broad-spectrum antibiotics, *Pneumocystis jiroveci* prophylaxis with cotrimoxazole, ganciclovir progressing to foscarnet due to proven resistant CMV strains and hyperimmune CMV-specific immunoglobulin (Cytogam®). Despite aggressive anti-viral therapy, it was impossible to fully control the CMV infection or the nephrotic syndrome that fluctuated in accordance with blood CMV titers that remained between 1 × 10^6^ and 4 × 10^6^ copies/ml despite aggressive treatment. Immunosuppressive therapy, first mycophenolate mofetil (MMF) later cyclosporine were initiated. She had an initial response to this treatment achieving reduction of proteinuria. Unfortunately, CMV infection could not be cleared and the nephrotic syndrome finally became unresponsive to treatment. In this clinical context stem cell transplant was not feasible. During this final end stage disease, she developed *Pneumocytis jiroveci* pneumonia and fungal lung infection (Figure 1E). Bronchoalveolar lavage was positive for *Pneumocystis jiroveci*, CMV and galactomannan. She progressed to multi-organ failure and died at 13 years of age, 1 year after her initial admission for nephrotic syndrome associated with systemic CMV infection.

**Figure 2:**
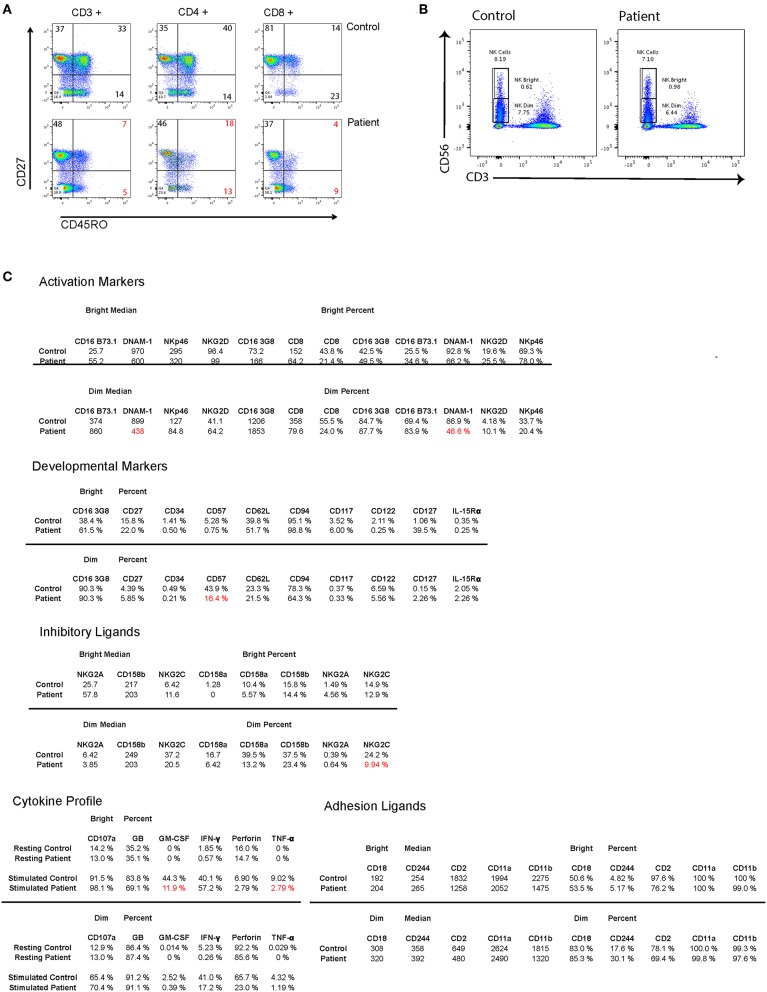
**(A)** Flow cytometry dot plots showing T cell memory subsets in total CD3^+^ T cells as well as in CD4^+^ and CD8^+^ subsets. **(B)** Flow cytometry dot plots showing patient and control NK CD56^+^ NK cells and gating on CD56^Dim^ and CD56^Bright^ NK cells. **(C)** 5 panels showing mean fluorescence intensity and percent of positive cells for different NK cell markers and receptors within CD56 ^Dim^ and CD56 ^Bright^ NK cells. Low percentages of cells compared to healthy donor are marked in red.

Heterogenic immune abnormalities may have contributed to patient clinical manifestations and susceptibility to chronic CMV infection. In order to understand these abnormalities, further phenotypic characterization of patient T cells and NK cells was performed post mortem from cryopreserved PBMC. In T cells a reduction in central memory and effector memory T cells in both CD4^+^ and CD8^+^ T cell compartments was observed (Figure 2A). Comprehensive NK cell phenotyping was performed as previously described (22). NK cell numbers and the proportion of CD56^bright^ and CD56^dim^ NK cells were within normal limits and similar to a healthy donor control (Figure 2B). There were no detected abnormalities in expression of NK cell receptors associated with adhesion, activation and inhibition, or NK cell development, and despite chronic CMV infection we did not observe expansion of NKG2C^+^ or CD57^+^ subsets as has previously been reported (23). Cytokine production was assessed after 5-h stimulation with PMA and ionomycin. While interferon gamma production was similar to control, a reduction in production of GM-CSF and TNFa was observed. CD107a mobilization, and perforin levels were comparable to control (Figure 2C).

Written informed consent was obtained from the parents of the patient for the publication of this case report and any potentially-identifying information/images.

## Discussion

Two members of the NF-κB transcription factor family, NF-κB1 and NF-κB2, are produced as precursor proteins, NF-κB1 p105 and NF-κB2 p100. Transcriptional regulation by NF-κB proteins involves canonical and alternative pathways that result in the nuclear translocation of the activated form of these proteins, p50 and p52 respectively. In the non-canonical pathway, the *NFKB2* transcripts are first translated into a p100 precursor protein that undergoes activation-dependent cytoplasmic proteolytic processing through NF-κB-inducing kinase (NIK) into the smaller active transcription factor p52. NF-κB2/p100 processing is stimulated by a subset of NF-κB inducers (lymphotoxin-β, B-cell activating factor and CD40 ligand) to regulate peripheral lymphoid organogenesis, B-lymphocyte differentiation and adaptive humoral immunity (24, 25).

As previously reported, mutations at the 3'end of the *NFKB2* gene, encoding the C-terminal region of the protein, both perturb immune and endocrine function resulting in central adrenocorticotropic hormone deficiency and CVID (10–13). Our patient carries a novel C-terminal mutation in NF-κB2 and presented with a clinical and immunological phenotype that very closely resembles other individuals with C-terminal mutations in this gene, including early onset combined immunodeficiency, alopecia and trachyonychia. Adrenal insufficiency was not clinically evident and was only diagnosed after the *NFKB2* mutation was identified.

**Figure 3:**
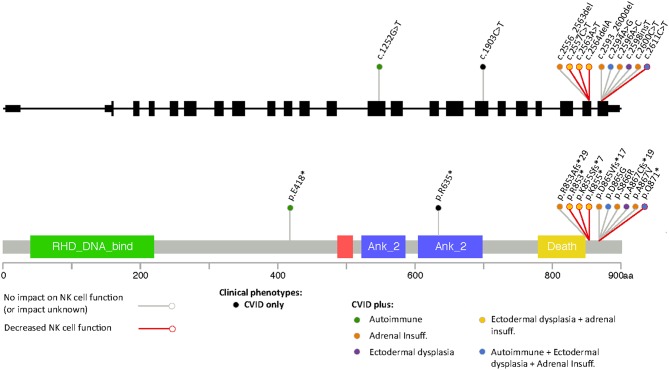
Representation of different variants in *NFKB2* that have been associated to disease color-coded with the different clinical manifestations including defects in NK cell function.

Most of the mutations associated with primary immunodeficiency that have been described for *NFKB2* are LoF mutations in the C-terminal domain, with variations at Arg853 being the most frequently reported (Figure 3). In most cases the nonsense/frameshift alleles with premature termination codons (PTC) have transcripts predicted to escape NMD and result in a truncated proteins that loose phosphorylation sites that are important for its interaction with NIK. As a result, p100 is not processed into p52. Two families with gain-of-function (GoF) alleles have been recently described, the mRNA transcribed from these disease-causing nonsense variants also seems to escape NMD and result in truncated proteins that, in contrast to C-terminal nonsense mutations, result in increased accumulation of p52 in the nucleus. Interestingly, for the E418^*^ variant, one asymptomatic family member carries the variant but has no truncated protein expression; suggesting mRNA degradation may be protecting him from disease (20). Our patient's stop gain mutation is also predicted to not render the mRNA unstable and subjected to NMD and thus the mutant transcript is likely to be translated and encode a C-terminal-aberrant protein due to the PTC (19). The Gln871 residue is immediately adjacent to the S870 phosphorylation site of NF-κB2 where p100 interacts with NIK to induce its processing into p52. Hence, it is likely that Gln871^*^ would interfere with p100 processing to p52 resulting in decreased p52 in the nucleus, thus resembling *Nfkb2* mutant mice and previously described patients with C-terminal missense mutations that compromise the NIK interaction region as well as nonsense and frameshift mutations that result in PTCs (Table 2, Figure 3) (13, 26).

In patients with C-terminal *NFKB2* mutations, the defect in the non-canonical NF-κB pathway invariably impairs B-cell function, resulting in hypogammaglobulinemia and poor antigen-specific antibody responses and presenting as CVID in early childhood (Table 2). Defects in peripheral T cells (12) as well as decreased natural killer (NK) cell function have also been described (15, 18).

NK cells are critically important for anti-tumoral and anti-viral defense and primary immunodeficiencies in which the main immunological defect is in NK cells (NK cell deficiencies) are clinically characterized by disseminated or recurrent viral infections due to CMV, herpes simplex virus (HSV), human papilloma virus (HPV), virus varicella zoster (VVZ) and Epstein Barr virus (EBV) (27). Five out of 6 NK cell deficiencies involve a defect in NK cell maturation.

NF-κB1 plays a role in NK cell maturation and effector function and patients with monoallelic mutations in NF-κB1 have functional NK cell defects that correlate with a susceptibility to viral infections mainly with viruses from the herpes family, including Epstein-Barr virus (EBV), varicella zoster and CMV (28). The role of NF-κB2 in NK cell function has not been clearly defined and an aberrant NK cell function does not seem to be a consistent feature of patients with C-terminal mutations (Figure 3). Thus far, it has been demonstrated in two previously published cases and ruled out in three additional patients (15, 18, 29). Some patients with C-terminal *NFKB2* mutations have presented with recurrent herpes labialis and systemic CMV or EBV infection but it remains unclear if these clinical manifestations occurred in the context of functional NK cell defects, although some had low NK cell numbers (Table 2) (10, 14, 20). Similar to our findings Lougaris et al. describe a patient with a C-terminal *NFKB2* mutation and functional NK cell deficiency in which NK cell phenotyping suggested intact NK cell maturation. In contrast to our observations, they report an increased expression of CD69 on CD56^dim^CD16^+^ NK cells, suggesting an activated steady state, however the viral status of this patient with regards to CMV and EBV that could have an impact in the expression of these markers was not reported (18). In healthy subjects, CMV infection drives an expansion of NKG2C^+^ NK cells (30); interestingly we did not observe an expansion of NKG2C^+^ or CD57^+^ NK cell subsets in our patient, despite chronic CMV infection.

The cytotoxic NK cell defect despite normal NK cell numbers identified in our patient is consistent and likely contributes to her susceptibility to invasive CMV infection, while further suggesting a possible role for NF-κB2 in NK-cell cytotoxic activity. A possible explanation for a functional NK cell defect could be the accumulation of unprocessed p100 in the cytoplasm that could potentially impair the canonical NF-κB pathway, but further investigation is needed to define the mechanism by which NF-κB2 deficiency may impact NK cell development, activation and function. The NK cell abnormality in patients with NIK deficiency had previously raised this question but those patients had defective canonical as well as non-canonical NF-κB activation (31). Functional T cell abnormalities identified in our patient, namely decreased proliferation to PHA and a reduction in CD8^+^CD45RO^+^ memory cells are similar to what has been described in one previously reported patient (12). We did not perform further T cell functional studies and it is unclear if these T cell abnormalities are a consistent feature of this disease. It is likely that both T cell and NK cell abnormalities contributed to an overwhelming CMV infection in this patient. Anti-cytokine antibodies were not measured in our patient but they have also been described in patients with mutations in *NFKB2*, these may be directed to type 1 interferons and also contribute to an impaired antiviral immunity (32).

The outcome and complications of patients with *NFKB2* mutations with or without functional perturbations of NK cells has not been compared. Importantly, this case highlights the risk of invasive viral infections including CMV in this disorder, and suggests that functional NK deficiency may play a role in this susceptibility and result in severe complications. We suggest that NK cell function should be assessed in all patients with mutations in the non-canonical NF-κB pathway. In those cases where NK function is affected, hematopoietic stem cell transplant may be appropriate despite the fact that NF-κB2 expression is not restricted to hematopoietic cells, since once CMV infection settles in, it becomes very difficult to clear and therefore reduces the chances for future successful stem cell transplantation.

Endocrine manifestations are more variable and expression of clinically overt ACTH deficiency involves about two-thirds of the cases and is usually an isolated form of hypopituitarism. However, growth hormone (GH) deficiency and hypothyroidism have also been described (15). Alopecia, trachyonychia and psoriatic dermatitis are also disease-associated features. Both endocrine and cutaneous manifestations are thought to be autoimmune mediated as NF-κB2 has been shown to play a role in central tolerance through an Aire-dependent pathway and in regulatory T cell homeostasis and suppressive function (15, 17, 33, 34). The underlying mechanism for the organ specificity of these autoimmune manifestations has not been fully elucidated.

Only one patient with *NFKB2* mutation has been previously reported to be associated with a benign nephrotic syndrome not related to CMV infection (15). The nephrotic syndrome in our patient was severe and unresponsive to immunosuppressive treatment. This is the first time an *NFKB2* variant has presented with long-lasting renal disease. The precise trigger for this manifestation is unknown. Interestingly, CMV PCR of the kidney biopsy was positive and her nephrotic range proteinuria fluctuated along with blood CMV titers. In periods of overwhelming CMV infection her nephrotic syndrome was most severe and unresponsive to treatment. CMV infection has been previously associated with nephrotic syndrome (35–38) supporting the role of CMV in the persistent proteinuria of our patient. Other possible causes of nephrotic syndrome described in patients with CVID are amyloidosis (39) and membranous nephropathy (40). An underlying autoimmune process cannot be ruled out, especially in the context of an *NFKB2* mutation, however the absence of autoantibodies and immune complex deposition in the kidneys argues against this possibility.

In conclusion, we report a patient with a novel mutation C-terminal in *NFKB2*, presenting as early onset CVID, ectodermal dysplasia and alopecia who had a functional NK cell defect and succumbed to overwhelming CMV infection and an associated nephrotic syndrome.

## Ethics Statement

This study was carried out in accordance with the recommendations of Institutional review board for Baylor college of Medicine and affiliated Hospitals with written informed consent from all subjects. All subjects gave written informed consent in accordance with the Declaration of Helsinki. The protocol was approved by The Baylor College of Medicine Institutional Review Board.

## Author Contributions

AA and MP wrote the manuscript. AV-H performed experiments and analyzed the data. EM and LF supervised analysis of NK cell studies. AA, ML, AK, and FC participated in the patient's clinical care. ZC-A, SJ, and IC performed and analyzed ES results. AR participated in coordination of sample analysis and Sanger sequencing confirmation. JL and JP supervised data interpretation of genomic studies. JL and JO were involved in study design and critically revised the manuscript. All authors reviewed the manuscript.

### Conflict of Interest Statement

The authors declare that the research was conducted in the absence of any commercial or financial relationships that could be construed as a potential conflict of interest.
